# Pediatric Philadelphia-Negative Myeloproliferative Neoplasms in the Era of WHO Classification: A Systematic Review

**DOI:** 10.3390/diagnostics13030377

**Published:** 2023-01-19

**Authors:** Abdulrahman F. Al-Mashdali, Mahmood B. Aldapt, Alaa Rahhal, Yousef M. Hailan, Israa Elhakeem, Elrazi A. Ali, Waail Rozi, Mohamed A. Yassin

**Affiliations:** 1 Department of Internal Medicine, Hamad Medical Corporation, Doha 3050, Qatar; 2 Department of Medicine, Unity Hospital, Rochester Regional Health, Rochester, NY 14626, USA; 3 Pharmacy Department, Hamad Medical Corporation, Doha 3050, Qatar; 4 Clinical Oncology, Hamad Medical Corporation, Doha 3050, Qatar; 5 One Brooklyn Health, Interfaith Medical Center, Internal Medicine Department, Brooklyn, NY 11213, USA; 6 National Center for Cancer Care and Research, Department of Oncology, Hematology and BMT Section, Hamad Medical Corporation, Doha 3050, Qatar

**Keywords:** myeloproliferative neoplasms, MPN, pediatrics, essential thrombocythemia, polycythemia vera, primary myelofibrosis

## Abstract

Background: Philadelphia-negative myeloproliferative neoplasms (MPN) are most prevalent in the older population (median age at the diagnosis is above 60 years) and rarely diagnosed in pediatrics. Thus, our knowledge about the clinical presentation, mutational status, and complications of MPNs in pediatrics is limited. Methods: The literature in English (PubMed, SCOPUS, and Google Scholar) was searched for studies, reviews, case series, and case reports of patients with Philadelphia-negative MPNs (including essential thrombocythemia, polycythemia vera, primary myelofibrosis, and profibrotic myelofibrosis) in the pediatrics age group (less than 18 years). Only studies that fulfilled WHO 2008 or 2016 criteria for MPNs were included. We aimed to describe the clinical characteristics, vascular and long-term complications, types of driver mutations, and treatment approaches in pediatric patients with MPNs. Results: We reviewed 33 articles of available published literature from 2008 to 2022 and collected data from a total of 196 patients of the pediatric population. Among the cohort of patients, 139 had essential thrombocythemia (ET), 20 had polycythemia vera (PV), and 37 had primary myelofibrosis (PMF). The median age at the time of diagnosis for each disease varied, with 8.8 years for ET, 10 years for PV, and 3.6 years for MF. There was a slight difference in gender prevalence between both gender groups and all three diseases. The presenting symptoms were not mentioned in more than 50% of studies. We found that JAK2 was the most prevalent among all mutations. Both bleeding and thrombosis were present equally in ET, with 9% of cases complicated by bleeding and 9% complicated by thrombosis. Hemorrhagic events did not occur in patients with PV; thrombosis in children with MF was also not found. The progression into AML occurred in two patients with PV and one with ET. Conclusion: Given the rarity of MPNs in pediatrics and their different characteristics compared with adults, we believe there is a need for unique diagnostic criteria to match the different molecular statuses in pediatrics. Based on our review, the incidence of MPN complications in pediatrics, including thrombotic events, hemorrhage, and leukemic transformation, differs from that in adults.

## 1. Introduction

Myeloproliferative neoplasms (MPN) are clonal hematopoietic disorders characterized by an overproduction of differentiated hematopoietic cells [1]. Traditionally, MPNs were known as “myeloproliferative disorders” (MPD); in 2008, World Health Organization (WHO) revised the classifications of MPDs and changed the name to MPNs, pointing to their malignant nature. The Philadelphia-positive (BCR-ABL1 positive) MPNs only includes chronic myeloid leukemia (CML), and Philadelphia-negative (BCR-ABL1 negative) MPNs include three major diseases: polycythemia vera (PV), essential thrombocythemia (ET), and primary myelofibrosis (PMF) [2]. The most common genetic mutations in Philadelphia-negative MPNs are JAK2 (PV = 100% of patients, ET = 50–60% of patients, PMF = 50–60% of patients), CALR (ET = 25–30% of patients, PMF = 30–35% of patients), and MPL (ET = 3–5% of patients, PMF = 5–10% of patients). However, around 10% of ET and PMF patients are “triple negative” for these mutations [1,3].

Philadelphia-negative MPNs are most prevalent in the older population (the median age at the diagnosis is above 60 years) and rarely diagnosed in pediatrics. It is estimated that MPNs are approximately 100 times more common in adults than children [1,4]. Historically, the clinical and hematologic findings in children with Philadelphia-negative MPNs were believed to be like those in adults. Accordingly, the same diagnostic criteria have been applied to adults and pediatrics. Also, the management approaches have been implemented based on adult treatment recommendations. However, recently, several studies have shown considerable differences between adult and pediatric MPNs. For instance, the frequency of JAK2 mutation in pediatrics with MPNs is less frequent than in adults. Also, complications (especially thrombotic events) in pediatrics with MPNs are less common than in adults [5,6].

This article reviewed the published cases of Philadelphia-negative MPNs in pediatrics (aged less than 18 years). We included all the articles published from 2008, when the WHO classification of tumors of the hematopoietic and lymphoid tissues was revised and updated. We aimed to describe the clinical characteristics, vascular and long-term complications, types of driver mutations, and treatment approaches in pediatrics with MPNs.

## 2. Methodology 

### 2.1. Literature Search Strategy 

**Figure 1:**
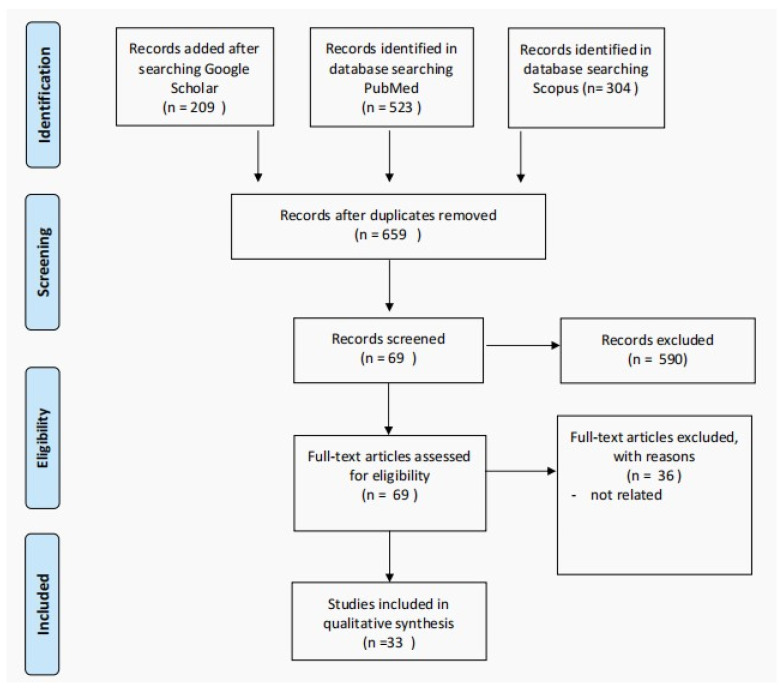
The PRISMA flow diagram detailing patients with the Ph-negative MPNs in pediatric age group.

We performed a systematic review following the Preferred Reporting Items for Systematic Reviews and Meta-Analyses (PRISMA) guidelines (Figure 1). The English-language literature (PubMed, SCOPUS, and Google Scholar) was searched for studies, reviews, case series, and case reports of patients with Philadelphia-negative MPNs (including essential thrombocythemia, polycythemia vera, primary myelofibrosis, and profibrotic myelofibrosis) in the pediatrics age group. We used the search terms: pediatrics or child with “essential thrombocythemia”, “primary myelofibrosis”, “polycythemia vera”, and “Profibrotic myelofibrosis”. The included studies’ reference lists were scanned for additional articles. The primary and secondary search processes included articles published from 2008 to 20 June 2022.

Inclusion criteria:
  - Pediatrics population (less than age 18 years).
  - Fulfilling WHO 2008 or 2016 criteria for Philadelphia-negative MPNs (ET, PV, Profibrotic MF, and PMF).
Exclusion criteria:
  - Age 18 and above.
  - Grey literature and narrative reviews.
  - Articles published before 2008 (not fulfilling WHO 2008 criteria).
Study selection

Two independent reviewers independently screened the titles and abstracts of the records, and papers unrelated to our inclusion criteria were excluded (Supplementary Material). Inter-rater disagreements were resolved following a discussion between the reviewers.

### 2.2. Data Extraction

Two reviewers extracted the following information: date of publication, country, study design, age, gender, age at diagnosis, disease, presenting symptoms, the presence of splenomegaly, family history of the same disease, initial CBC, the presence of specific mutation (JAK2, CALR, MPL), disease complications, disease progression, and the clinical outcome. All the extracted data are mentioned in Table 1.

## 3. Results

### 3.1. Clinical Characteristics

We reviewed 33 articles from the available literature published during the period 2008 to 2022 and collected data on a total of 196 patients of the pediatric population; 139 had essential thrombocythemia (ET), 20 had polycythemia vera (PV), and 37 had primary myelofibrosis (PMF) [3,6,7,8,9,10,11,12,13,14,15,16,17,18,19,20,21,22,23,24,25,26,27,28,29,30,31,32,33,34,35,36,37]. The patients’ clinical and biological characteristics are addressed in Table 1 and Table 2. The median age at the time of diagnosis for each disease varied, with 8.8 years for ET, 10 years for PV, and 3.6 years for MF. There was a slight difference in gender prevalence among all three diseases. The ET group showed a slight female predominance, with 53% female; in contrast, the PMF group showed a male predominance, with 68% male. There was no difference in gender prevalence in the PV group. 

### 3.2. Biological Characteristics

The mean white blood cell count (WBC) for ET patients was 13.2 × 109/L, for PV patients 10.7 × 109/L, and for PMF patients 21.6 × 10/L. The mean hemoglobin was 13.1 g/dL in ET patients, and the PMF patients had the lowest hemoglobin levels. Both ET and PV patients had significant thrombocytosis. We have addressed the parameters in more detail in Table 1 and Table 2.

### 3.3. Molecular Analysis

We found that JAK2 mutation was the most prevalent among all mutations, being present in 28 (20%) of the ET patients and 12 (60%) of the PV patients (9 had JAK2 V617F mutation and 3 had JAK2 exon 12 mutation). CALR and MPL were detected in ET, with MPL being more frequently detected, in 23 (17%) cases, while CALR was detected in only 8 (6%) cases. A total of 25 (18%) of the ET cases and 2 (6%) of the PMF cases were triple negatives for mutations.

### 3.4. Complications 

#### 3.4.1. Thrombotic and Hemorrhagic Events

Both bleeding and thrombotic events were present equally in ET, with 13 (9%) cases developing bleeding and 13 (9%) developing thrombosis. Thrombosis occurred in 2 (10%) of the PV cases. However, hemorrhagic events were not seen in patients with PV. In PMF patients, one occurrence of bleeding was reported (a 6-year-old male who died from an intracranial hemorrhage). No thrombotic event was found in the PMF cases. Details about the site of thrombosis and hemorrhage are mentioned in Table 1.

#### 3.4.2. Disease Progression 

Progression into acute myelogenous leukemia (AML) or overt MF is among the most serious complications of MPN. Only one patient (5%) with PV progressed into MF. Of those who had ET, 14 patients (10%) progressed into MF. The progression into AML was less common, with only two patients with PV and one with ET having progressed to AML.

#### 3.4.3. Treatment

Our review of published articles encountered several treatment approaches for each disease; however, the rationale behind why some agents were chosen over others was not widely discussed. Treatment and further details are described in Table 1 and Table 2. Aspirin was the most used treatment in ET patients, in 57 (41%) cases, followed by hydroxyurea in 30 (23%) patients. None of the ET cases underwent bone marrow transplantation, and around a quarter received no treatment. Interferon was the most used treatment in PV patients, in 9 (45%) cases, followed by venesection in 6 (30%) patients. Aspirin was used in only 3 (15%) of the PV patients. As with those with ET, 30% of the cases did not receive treatment. Only two patients received hydroxyurea; however, both cases were in combination with venesection. A total of 12 (32%) of the PMF patients did not receive treatment; the mainstay treatment was bone marrow transplant, in 13 (35%) cases.

## 4. Discussion

### 4.1. Epidemiology

MPNs in pediatrics are not well described in the literature, with little known about the prevalence, presentation, prognosis, and treatment. MPNs are far less common in children than adults, and our current review focused on Philadelphia-negative MPNs. In the United States, PV in adults has the highest incidence rate among MPNs, with 10.9 new cases per 1 million population per year. In contrast, the incidence of ET is 9.6 new cases per 1 million population per year, and PMF is the least frequent, with an incidence rate of 3.1 new cases per 1 million population per year [38]. The incidence of MPNs in pediatrics is much lower, with 0.09 new ET cases per 1 million population per year in children [39]. At the same time, PMF is very rare in children, and because of the rarity of the disease, precise epidemiological data are lacking, with data consisting mainly of case reports and series [37]; the same is true of PV. Therefore, the exact incidence is unknown, with estimates of two new cases per 10 million persons under 20 years old per year [40]. In our review of 196 pediatric cases with Philadelphia-negative MPNs, consistent with old reports, ET was the most prevalent (approximately 70% of the cases). In a cohort of 444 patients with MPNs diagnosed before 25 years of age from 15 different countries, ET was the most common (71.6%), far ahead of PV (18.2%) and other MPNs (10.1%) including PMF and unclassified MPNs [41].

### 4.2. Polycythemia Vera

A large international study included 1545 cases of PV in adults, based on the 2008 WHO diagnostic criteria, and had a median age of 61 years. The most common presentations were palpable splenomegaly, pruritus, and vasomotor symptoms, found in about a third of the patients. Venous thrombosis affected 7.4%, while arterial thrombosis was more common and affected 14%. On the other hand, hemorrhage was complicated in 4.3% of the cases, and leukemic transformation was found in 3% of the cases. In our review, the presence of splenomegaly was not mentioned in most cases, yet around a third of the reported data have splenomegaly, matching the percentage found in adults [41]. On the other hand, hemorrhage was not observed in pediatrics, while thrombosis was complicated in 2 (10%) cases, and leukemic transformation was documented in 2 (10%) cases. The number of PV cases in this review was small, with only 20 reported cases, so it was challenging to reach conclusions, yet the presentation and complications between adults and pediatrics were comparable, apart from a possibly higher chance for AML transformation in the pediatric population.

In this pediatrics review, JAK2V617F was found in 45% of the cases, and JAK2 exon 12 in 15%. This observation was also documented in a retrospective study of 11 pediatric patients with PV, where JAK2V617F was present in only 27% of cases [42]. In another cohort of 81 PV patients younger than 25 years, JAK2V617F was detected in 86.4% and JAK2 exon 12 in 6.2% [41]. Such a finding points out the need for unique diagnostic criteria for PV in pediatrics to match the different molecular statuses in pediatrics with PV. In the WHO diagnostic criteria for PV in adults, the presence of JAK2 mutation is essential and one of the major criteria for diagnosing PV, which might not be applicable in the pediatric population.

In this review, interferon-alfa (IFN-alfa) was the preferred cytoreductive treatment and was used in nine (45%) cases, followed by hydroxyurea (HU) in only two (10%) cases. In adults, HU and IFN-alfa are first-line cytoreductive therapy in high-risk PV [43]. HU is the preferred agent in adults because of its efficacy, lower cost, ease of administration, and low toxicity profile [44]. The possible use of IFN-alfa as the preferred cytoreductive agent in the pediatric population may be justified, and this is consistent with ELN’s suggestion to use HU with caution in patients <40 years [44]; the reasons for this preference include the possibility of achieving cytogenetic remission with IFN-alfa and the teratogenicity of HU [45,46]. Although all adult patients with PV should be treated with venesection to maintain hematocrit of less than 45% and low-dose ASA [44], in this review, the use of venesection and ASA in pediatric patients with PV was less common, with ASA being used in only three (15%) patients and venesection in six (30%) patients. In a cohort of patients with PV and <25 years of age, cytoreductive drugs were given to 71.6% of PV patients [41]. Finally, hematopoietic stem cell transplant (HSCT) is usually used in young patients with poor prognosis; however, it is not well studied in pediatrics with PV. It was only used in one patient in this review, resulting in complete remission and no signs of transplant-related morbidity after 78 months of follow-up [46]. Although all modalities of PV treatment used in adults have been applied in pediatrics, including phlebotomies, erythrocytapharesis, ASA, and HU [6], the safety and efficacy of such treatments are not well described, and HSCT remains the only curative treatment; further studies in this field are needed to address an unmet need. The same goes for prognosis; long-term survival is unknown in pediatrics.

### 4.3. Essential Thrombocythemia

A single-institute retrospective study showed that around 45.3% of adult patients with ET are asymptomatic at the diagnosis [47]. Our review has 139 pediatric ET cases; 36% were asymptomatic at presentation, a number lower than that observed in adults. The data for the presence of splenomegaly were not documented in around 50% of the cases; nevertheless, in the cases where splenomegaly was documented, 24 out of 71 (33.8%) patients had splenomegaly, a number matching the percent of splenomegaly in adults. While the incidence of bleeding in pediatrics matched that of adults, thrombosis was less common than in adults. In a cohort of ET patients younger than 25, thrombosis occurred in 8.5% of the patients, nearly matching the percentage in our review [41]. The lower incidence of the JAK2V617F mutation in pediatrics with ET may play a part in the lower risk of thrombosis [48]. MPN thromboinflammation also plays an essential role in the pathogenesis of venous thromboembolism. JAK2V617F was demonstrated to activate several integrins on mature myeloid cells, which increase adhesion and drive venous thrombosis in murine models [49]. The same scenario may apply to the reactive oxygen species (ROS), which have a significant role in the MPNs, where clonal cells produce excess ROS, creating a vicious cycle in which ROS activate proinflammatory pathways, which create more ROS [50]. Venous thromboembolism (VTE) formation is affected by ROS through regulation of the coagulation, fibrinolysis, proteolysis, and the complement system, as well as regulating effector cells such as myeloid cells, platelets, endothelial cells, and fibroblasts [51].

A cohort of 896 patients with MPNs was screened for mutations in JAK2V617F, MPL, and CALR. A total of 311 patients had ET; of those, JAK2V617F was the most common mutation (59%), followed by CALR mutation (25%), and the least common was MPL mutation (3.5%). A total of 12.2% of the cases had JAK2/MPL/CALR wild type [52]. In our review, JAK2V617F was also the most common mutation and was reported in 28 (20%) patients, while MPL mutation was documented in 23 (16.5%) patients, and the least frequent mutation was CALR in 8 (6%) patients. In contrast, 25 (18%) patients had JAK2/MPL/CALR wild type, and the rest were identified as either JAK2/MPL wild type or JAK2V617F wild type. Although JAK2V617F was still the most common mutation in pediatrics, it was found in only 20% of the cases, and more patients had no mutations identified than was the case with adults [53]. Giona et al. found JAK2V617F mutations in 40–50% of sporadic pediatric cases, and patients with hereditary ET had no JAK2 mutations [43]. Another cohort found JAK2V617F in 48.5% of young ET patients [41]. These findings raise several questions involving the need to screen for non-JAK2V617F variants in pediatric MPNs [49]. Furthermore, next-generation sequencing (NGS) studies may play a role in the diagnosing and prognosis of MPNs in pediatrics, as somatic mutations on ASXL1 and TET2 might be playing a role in triggering MPNs [54,55]. 

As in PV, high-risk patients with ET should be treated with cytoreduction therapy. Hydroxyurea is the first-line treatment, as it reduces thrombotic complications compared to no treatment [52,56]. However, for patients who are intolerant to hydroxyurea, or when the disease is resistant to hydroxyurea, treatment can be changed to anagrelide [44] and oral imidazoquinazoline derivative that decreases circulating platelets by reducing both megakaryocyte hyperproliferation and differentiation [57]. In the noninferiority ANAHYDRET study, anagrelide was found to be non-inferior to HU [58]; nevertheless, its cardiac toxicity has limited its use [59]. IFN-alfa can also be used but should be reserved for young female patients because there is no evidence that it can improve overall survival. However, the safety profile for IFN-alfa makes it an option for patients who are intolerant or resistant to HU and anagrelide [60].

In our cohort of pediatric ET patients, eighty-five patients received treatment; 57 (67%) of these patients received aspirin. The most used cytoreductive treatment was HU, in 30 (35%) patients, followed by anagrelide in 15 (18%) patients, and 9 (10.5%) patients received IFN-alfa; this is consistent with the guidelines to treat adults with ET. In another cohort, 68.2% of young patients with ET were prescribed cytoreductive drugs. Interestingly, HU was used more liberally in ET than was the case with PV; the reason behind this is not apparent and reasons like ET patients being more symptomatic and having more complications than PV are possible but cannot be concluded from our collected data. We again face the same limited data when addressing treatment options, outcomes, and prognosis for ET in children, and a standard treatment approach is not available. Furthermore, age has been considered as one of the high-risk factors per definition along with JAK2 mutations; however, age is not applicable in the pediatric population, and JAK2 mutation prevalence is less common; this would affect the choice of treatment used in pediatrics, and accordingly, a dedicated risk stratification model is needed. In a cohort of 444 young MPN patients, conventional risk scores did poorly in predicting complications and mortality; instead, splenomegaly and hyper-viscosity symptoms better predict thrombosis and leukemic transformation [41].

### 4.4. Primary Myelofibrosis

PMF is the least common MPN in adults, and the disease is rare in the pediatric population. PMF in adults is mainly presented with constitutional symptoms, in 34% of the patients, and palpable splenomegaly in 31% [61]. Our cohort included 37 pediatrics with PMF; 16 (43%) patients were symptomatic, including 22 (59%) patients with palpable splenomegaly. Based on our review, PMF cases in pediatrics are more common than PV cases, compared to adults, where PMF is the least common. Data for pediatrics with PMF is scarce; based on this sample of 37 patients, the percentage of symptomatic patients is higher than adults, but unlike adults, most pediatrics are presented with symptoms of cytopenia rather than constitutional symptoms, and the same was true for palpable splenomegaly where it was more reported compared to adults.

In adults, the most common mutations in PMF are JAK2V617F, in 61% of cases, followed by MPL mutations in 8% of the cases [61]. CALR mutation was found in 88% of adult patients with both JAK2/MPL negative disease [52]. On the other hand, the pediatrics with PMF had an entirely different mutation profile, with none of the patients in our data having JAK2V617F or MPL mutations. Nevertheless, one patient had a CALR mutation. Sequence analyses for JAK2V617F and MPLW515L mutations were performed on bone marrow samples from 17 pediatric patients with PMF; none of the patients had positive results [37]. The incidence of post-diagnosis fatal and nonfatal thrombosis in 707 adult patients with PMF was recorded in 51 (7.2%) patients [62]. On the other hand, in a retrospective analysis of 205 adult patients with PMF and known JAK2V617F mutations, post-diagnosis thrombosis was registered in 22 (10.7%) patients [63]; this indicates that JAK2 is a risk factor for thrombosis in patients with PMF. Interestingly, in our collected data, none of the patients with PMF developed thrombosis, and none had JAK2 mutations. Furthermore, 7% of adult patients have documented leukemic transformation [61]; in contrast, no leukemic transformation was recorded in our collected data. 

The only curative treatment approach for PMF is HSCT. Other treatment options are also used in PMF, including erythropoiesis-stimulating agents, corticosteroids, androgens, danazol, thalidomide, and lenalidomide. In contrast, hydroxyurea is used to treat symptomatic splenomegaly and symptoms of symptomatic thrombocytosis and leukocytosis. Other approaches to treat splenomegaly include splenic radiation, which has only transient benefits [43]. Splenectomy is indicated in symptomatic portal hypertension, drug-refractory splenomegaly that is painful or associated with cachexia, and blood transfusion-dependent anemia; splenectomy carries a perioperative mortality risk of 5-10% and perioperative complications in approximately 50% of patients [60,64]. Recently, ruxolitinib, a JAK2 inhibitor, has been approved by the United States Food and Drug Association (FDA) and the European Medicines Agency (EMA). Interestingly, its efficacy is independent of JAK2 mutation status [65] and it results in a significant reduction in splenomegaly and relief of symptoms [66]. Of note, the evidence for improved survival in PMF patients treated with ruxolitinib is moderate to poor [67].

In this review, about a third of patients did not receive any treatment, and 13 (35%) patients underwent HSCT. PMF in pediatrics is very rare; it presents differently than in adults, and those common mutations seen in adults, mainly JAK2 and MPL, are not seen in pediatrics. Furthermore, the third major criterion of the WHO diagnostic criteria for PMF, which is the demonstration of a disease-specific mutation, cannot be applied in pediatrics because the mutations are rare and even absent in some reports. Hereditary PMF may play a role in PMF genetics (seven patients in our series have a family history of PMF). 

Young adult patients (<60 years) with PMF and Dynamic International Prognostic Scoring System-plus (DIPSS-plus) with low or intermediate-1 risk have a good prognosis in terms of overall survival [61]; this may also translate to the pediatric population with PMF. However, prognostic scoring systems like DIPSS cannot be applied in pediatrics simply because they depend on age and cytogenetics as prognostic factors. Furthermore, AML transformation and other complications like thrombosis are rare in pediatrics. Additionally, cases with rapid progression and death have been reported, especially in familial cases [21]. 

Next-generation sequencing (NGS), or massive parallel sequencing, was developed in the last decade and permits concurrent sequencing of millions of DNA fragments without previous sequence knowledge [68]. In the past decade, there has been an expansion in knowledge of the complex mutational background of MPNs, which has led to a distinctive change in the diagnosis, classification, and treatment of MPNs. Non-driver somatic mutations associated with the pathogenesis of MPNs belong to a variety of functional classes, including mutations in the epigenetic regulator genes [69]. Based on the revised 2016 WHO criteria, in triple-negative MPN patients, testing for the most frequent additional mutations using NGS may be valuable to determine the clonal nature of the disease and add to the morphological criteria. Interestingly, non-driver mutations might precede or follow the gaining of driver mutations during the clonal evolution from early to blast phase MPNs [70]. In an NGS study on 197 patients with MPNs, mutations in TET2 or DNMT3A were often present in the early clones acquired before JAK2V617F, while mutations in ASXL1, EZH2, or IDH1/2 were often acquired after JAK2V617F [71]. With NGS becoming more used in clinical practice, the integration of clinical data with genomic profiling data for MPNs will allow tailored treatment modalities for patient management and personalized prediction of outcomes [69].

The main limitation of our systematic review was that the patients’ data were mainly obtained from case reports and series, so the long-term outcomes for those patients could not be appropriately assessed. Also, the number of included cases was limited due to the rarity of MPNs in pediatrics, especially those diagnosed after 2008. Lastly, there were numerous missing data in those case reports, including but not limited to the presenting symptoms, mutation status, and follow-up, which significantly restricted our review.

## 5. Conclusions

MPNs are being increasingly found in the pediatric population, though pediatric MPNs are still rare. Available evidence for the diagnosis and management of MPNs in pediatrics is mainly based on the experiences of the adult population. Studies on pediatric MPNs are largely retrospective, with short follow-up duration. Given the rarity of MPNs in pediatrics and their different characteristics compared with adults, we believe there is a need for unique diagnostic criteria to match the different molecular statuses in pediatrics. Additionally, MPN conventional risk scores performed poorly in predicting complications and mortality in pediatrics, and a dedicated risk stratification model for pediatrics is needed. Based on our review, the incidence of MPN complications in pediatrics, including thrombotic events, hemorrhage, and leukemic transformation, differs from that in adults. Nevertheless, cohorts with extended follow-ups are necessary to detect the true incidence of these complications in pediatrics.

## Figures and Tables

**Table 1:** Characteristics, outcomes, and treatment of patients with essential thrombocythemia, polycythemia vera, and myelofibrosis [3,6,7,8,9,10,11,12,13,14,15,16,17,18,19,20,21,22,23,24,25,26,27,28,29,30,31,32,33,34,35,36,37].

Author/Year of Publication	Gender	Disease	Symptom(s)	Splenomegaly	Comorbidities	Family History	Labs (Range)	Mutation	Complications	Treatment	Follow Up	Comments
	Age (Range)											
Robins [1], 2008	M	ET	Asymptomatic	Y	Y	Y	PLT 900	Double negative	None	ASA HUS Anagrelide	NA	
	(2 y)											
Nakatani [2], 2008	3 M and 3 F	ET	1: Headache 5: Asymptomatic	2: Y 4: N	N	N	WBC 6.3–86 PLT 687–2709 Hb 11.7–15.2	3: JAK2V617F	None	1: HU 6: None	8–47 m	
	(2 m to 14 y)											
Kurosawa [3], 2009	1 F	ET	Headache	Y	N	N	WBC 10.5 PLT 680 Hb 12.5	JAK2V617F	Thrombosis (CVT)	HU	24 m	
	(6 y)											
Domm [4], 2009	2 M	MF	1: Jaundice 1: Infection (abscess)	Y	1: Premature delivery 1: Recurrent pneumonia	1: None 1: NA	1: WBC 45.2, PLT 90, Hb 5.7 1: ANC 70, PLT180, Hb 8	NA	NA	2: BMT	29–48 m	
	3–9 m											
Teofili [5], 2010	11 M and 10 F	ET	NA	11: Y 10: N	Anemia: 7 CKD: 1	Y	WBC 4.2–20.5 PLT 317–1726 Hb 10–15.5	MPL	8: Progressed to MF	7: ASA 4: ASA and HU 1: ASA, HU, and INF 8: None	12–240 m	
	Median:18 years (Range, 1–76)											
Aviner [6], 2012	1 M and 1 F	ET	1: Abdominal pain 1: Asymptomatic	N	N	N	WBC 10.5–17.3 PLT 1644–4200 Hb 11.4–123	JAK negative	None	ASA	48–204 m	Spontaneous remission
	4–13 y											
Dua [7], 2012	1 M and 1 F	ET	Fever	N	N	N	WBC 9.9–14.4 PLT 1119–1630 Hb 12–13.9	1: JAK2V617F	None	HU: 1 HU and ASA: 1	17–20 m	
	5–10 y											
Khan [8], 2012	1 F	ET	Headache	N	N	N	WBC 9 PLT 943 Hb 12.5	JAK negative	Thrombosis (CVT)	VKA and ASA	NA	
	13 y											
Ismael [9], 2012	7 M and 6 F	9: ET 4: PV	NA	2: Y(ET) 1: Y(PV) N: 10	NA	N	WBC 5.8–19.4 PLT 106.4–956 Hb 11.7–21.8	3: JAK2V617F (ET = 1, PV = 2) 10: Double negative	NA	6: ASA (ET cases) 6: None 1: Phlebotomy (PV)	12–240 m	
	(Median age 10 years; range 1.5–15 years)											
Nirupam [10], 2012	1 F	MF	Fever, pallor	Y	Β-thalassemia intermedia	N	WBC 25 PLT 148 Hb 2.5	NA	Progressed	HU	1.5 m	
	10 y											
DeLario [33], 2012	12 M and 7 F	MF	NA	12	NA	NA	18: Hb < 11 7: ANC < 1.5 17: PLT < 150	None	None	5: Spontaneous recovery 9: HSCT 8: Death (4 after HSCT)		
	0–17 year											
Shaikh [11], 2012	1 F	MF	Fever, pallor, abd distension	Y	None	N	WBC 17.5 PLT 35 Hb 7.4	JAK negative	Progressed	BMT	18 m	BMT complicated by acute GVHD, engraftment syndrome, hepatic venoocclusive disease
	11 m											
Slone [12], 2013	2 M and 1 F	2: MF 1: ET	1: asymptomatic (MF) 1: Back pain (MF) 1: Headache and blurred vision (ET)	N	NA	NA	Patient 1 (hemoglobin 5.5 g/dL) Patient 2 (maximum platelet count 4,000,000/mL) Patient 3 (PLT ranged 845,000 to 1,464,000/mL, WBC 12,109/L, hemoglobin 15.6 g/dL)	1: JAK2V617F (ET) 2: Double negative	1: Progressed (MF) 1: CVT (ET patient)	1: None (MF) 2: HU (MF and ET)	12–72 m	MF cases had spontaneous remission in one patient
	2–12–15 y											
Farruggia [13], 2013	1 M		ET	Pain, seizure	Y	ADEM	N	WBC 9.7 PLT 1478 Hb 11.6	MPL	None	ASA	24 m
	4 y											
Stepensky [14], 2013	4 M and 1 F	MF	Recurrent infections and abscesses	2: Y 3: N	NA	Y	ANC 100–300 PLT 32–260 Hb 7.3–8.6 Average at presentation (average Hb 7.84) (average ANC 190) (average PLT count 148.4)	N/A	5: Progressed	None	1 wk to 24 m	2: Died 2: Posted for BMT 1: Had BMT
	1–5 m											
Valfaie [15], 2013	1 M	ET	Asymptomatic	Y	N	N	PLT 303 × 109 to 2131 × 109/L	Double negative	None	ASA, Anagrelide, HU	12 m	PLT 580
	10 y											
Saksena [16], 2014	1 M	MF	Fever, pallor	Y	Pulmonary TB	N	WBC 3.6 PLT 64 Hb 4.1	NA	Progressed	None	NA	
	10 y											
Togoz [17], 2015	1 F	ET	Fatigue, abdominal pain	Y	NA	N	PLT 1300 range (1000 and 1400)	MPL	Thrombosis (Hepatic vein, IVC)	HU PLT apharesis	NA	Underwent liver transplantation
	15 y											
Wigton [18], 2016	1 F	ET	Pain (abdominal)	Y	N	N	PLT 415	JAK2V617F	Thrombosis (Hepatic veins)	Anagrelide, HU, plasmapharesis	108 m	Underwent liver transplantation
	12 y											
Kucine [19], 2016	5 F	ET	3: Headache 1: Bleeding (nose) 1: Pruritis	NA	NA	N	PLT 800–2800	3: JAK2V617F 2: Triple negative	1: Bleeding * 4: None	5: HU	NA	
	5–19 y											
Mazher [20], 2017	1 M	MF	Fever, bleeding (petechiae)	Y	NA	NA	NA	Triple negative	Bleeding (ICH)	None	NA	Died due to ICH
	6 y											
Khan [21], 2017	1 F	MF	Asymptomatic	No	Neurocognitive disorders	Y (leukemia)	WBC 11.9 PLT 67 Hb 8.4	Triple negative	None	None	8 m	
	14 y											
Aladily [22], 2017	1 F	ET	Headache	N	N	N	PLT 600	Triple negative	Bleeding (epistaxis)	ASA, HU	96 m	
	2 y											
Schneider [23], 2019	1 F	ET	Headache, visual impairment, extremity pain	Y	NA	N	PLT 2373	Triple negative	Bleeding (epistaxis, menorrhagia)	ASA, INF, HU	30 m	Underwent PLT apheresis
	14 y											
Tafesh [24], 2019	2 M and 1 F	1: PV 2: ET	1: Dizziness 1: Headache, paresthesia, left-sided weakness 1: Abdominal pain, UGI bleeding	1: Y	1: Portal HTN	NA	2: PLT 641–1123 1: Hb 18.6	1: JAK2V617F(ET)	2: CVT (1 PV, 1 ET) 1: UGI Bleeding (1 ET) and thrombosis^1^	1: ASA 1: HU 1: Venesection	NA	
	11,15,17 y											
Assaf [25], 2020	5 M and 7 F	ET	3: Headache 1: Blurred vision 1: Seizure 1: Erythromelalgia 1: Corpus luteum bleeding 5: Asymptomatic	NA	NA	NA	WBC 7.3–25.3 PLT 717–4.200 Hb 11.3–15.6	6: JAK2V617F 2: CAL-R (type 1 and 2) 4: triple negative	2: Thrombosis (TIA) 1: Thrombosis (CVC) 1: Bleeding (Menometrorrhagia) and thrombosis (TIA) 1: Bleeding (IAB)	7: ASA 4: HU 6: None	5–108 m	
	1–14.5 y											
Jeffrey [26], 2020	2 M	MF	Pruritic rash	Y	N	N	PLT 11–36	NA	2: Death	1: BMT	2.5–3 m	
	1 wk (both)											
Dmitrii [27], 2020	12 M and 8 F	ET	7: Headache 3: Bleeding (nasal) 10: Asymptomatic	10: Y 10: N	NA	NA	PLT 561-	3: JAK2V617F 4: CALR 13: triple negative	3: Bleeding * 3: Progressed to MF	NA	NA	
	0.6–16 y											
Sarah [28], 2021	1 F	PV	Pain (leg)	NA	NA	NA	WBC 8.77 PLT 365 Hb 13.7	JAK2 exon-12	NA	ASA, INF, venesection	15 M	
	2 y											
Benjamin [29], 2021	1 F	PV	Fever, pruritis, erythroderma	Y	NA	N	WBC 11 PLT 119 Hb 19.2	JAK2V617F	NA	HU, venesection	NA	
	5 y											
Arumugom [30], 2021	1 F	PV	Asymptomatic	Y	N	N	PLT 1116 Hb 19.3	JAK2V617F	None	ASA	24 m	
	12 y											
Hisachi [31], 2021	26 M and 24 F	5: PV (4 M and 1 F) 44: ET (21 M and 23 F) 1: PMF (1 M)	NA	NA	NA	4: Y *	WBC 4.5–18 PLT 20–1432 Hb 8.8–24.5	9: JAK2V617F (ET cases) 1: CLAR (not specified case)	2: Leukemia (1 PV and 1 ET) 4: MF (1 PV, 3 ET) 4: Bleeding * (4 ET) 3: Thrombosis * (3 ET)	21: ASA(ET) 12: Anagrelide (ET) 7: HU (6 ET, 1 PV) 2: Phlebotomy (PV) 14: None (11 ET 3 PV)	0.2–237.1 m	
	0.0–15 y											
Nicole [32], 2021	5 M and 8 F	7: PV (3 M and 4 F) 6: ET (2 M and 4 F)	NA	6: Y	NA	NA	9: PLT > 1000 4: NA	6: JAK2V617F (1 ET and 5 PV) 2: JAK2 exon-12 (2 PV) 1: CAL-R (1 ET) 4: triple negative (4 ET)	1: Upper GI bleed (PV) 2: PE (PV, ET) 1: CVT (ET)	3: INF only 10: HU and INF	10–168 m	
	2–16 y											

M: Male. F: Female. y: Year. ET: Essential thrombocytosis. PV: Polycythemia vera. MF: Myelofibrosis. MDS: Myelodysplastic syndrome. Y: Yes. N: No. PLT: Platelets. Hb: Hemoglobin. JAK-2: Janus Kinase 2 gene. MPL: Myeloproliferative leukemia protein (thrombopoietin receptor). CAL-R: Carcinogenic mutated form of the calreticulin gene. CVT: Cerebral venous thrombosis. TIA: Transient ischemic attack. DVT: Deep venous thrombosis. NA: Not available. HU: Hydroxyurea. ASA: Acetylsalicylic acid. m: Month. wk: Week. BMT: Bone marrow transplant. CVC: Central venous catheter. IAB: Intra-abdominal bleeding. PE: Pulmonary embolism. *: Details not available. Thrombosis^1^: Portal, hepatic, and splenic vein thrombosis as well as coronary artery thrombosis.

**Table 2:** Characteristics, outcomes, and treatment of patients with essential thrombocythemia, polycythemia vera, and myelofibrosis.

Characteristic	ET (*n* = 139), *n* (%)	PV (*n* = 20), *n* (%)	MF (*n* = 37), *n* (%)
Age at diagnosis (years) ^Ѳ^	8.8 ± 4.9	10.0 ± 4.8	3.6 ± 5.8
Gender			
Male	65 (47)	10 (50)	25 (68)
Female	74 (53)	10 (50)	12 (32)
Symptomatic			
Yes	39 (28)	3 (15)	16 (43)
No	50 (36)	5 (25)	2 (6)
Not available	50 (36)	12 (60)	19 (51)
Splenomegaly ^§^			
Yes	24 (18)	3 (21)	22 (59)
No	47 (35)	5 (36)	14 (38)
Not available	62 (47)	6 (43)	1 (3)
Family history	27 (19)	1 (5)	7 (19)
Comorbidities	4 (3)	0 (0)	5 (14)
Labs ^Ѳ^			
White blood cells (×10^9^/L)	13.2 ±10.7	12.8 ± 4.8	21.6 ± 13.4
Hemoglobin (g/dL)	13.1 ± 1.2	18.0 ± 2.5	6.6 ± 1.9
Platelet count (×10^9^/L)	1301.7 ± 814.5	609 ± 413	360 ± 1010
Complications			
Bleeding	13 (9)	0 (0)	1 (3)
Thrombosis	13 (9)	2 (10)	0 (0)
Progressed to MF	14 (10)	1 (5)	12 (32) *
Progressed to AML	1 (1)	2 (10)	0 (0)
Mutation			
JAK2 V617F JAK2 exon 12	28 (20) 0	9 (45) 3 (15)	0 (0) 0(0)
CALR	8 (6)	0 (0)	1(3)
MPL Triple negative	23 (17) 25 (18)	0 (0) -	0 (0) 2(6)
Treatment			
Aspirin	57 (41)	3 (15)	0 (0)
Anagrelide	15 (11)	0 (0)	1 (3)
Hydroxyurea ^‡^	30 (23)	2 (10)	2 (6)
Interferon	9 (6)	9 (45)	0 (0)
Venesection	3 (2)	6 (30)	0 (0)
Bone marrow transplantation	0 (0)	1 (5)	13 (35)
No treatment	34 (24)	6 (30)	12 (32)
Not available	20 (14)	0	9 (24)
Follow-up (months) ^Ѳ^	61 ± 61	49 ± 25	20 ± 20

ET: Essential thrombocythemia; PV: polycythemia vera; MF: myelofibrosis; AML: acute myeloid leukemia. ^Ѳ^ Values reported as mean ± standard deviation and [31,32,33] were excluded as they reported the values in range. ^§^ Six patients were reported to have splenomegaly by Kucine N et al. [32]; however, the disease was not specified for them; hence, we removed them from both ET and PV; total number of ET cases for splenomegaly = 133, and for PV = 14. * Progressed to advanced stages of MF (MF 2 and MF 3). ^‡^ Seven patients were reported to receive hydroxyurea by Kucine N et al. [32]; however, the disease was not specified for them; hence, we removed them from both ET and PV; total number of ET cases who received treatment = 133, and for PV = 13.

## Data Availability

All data derived from this study are presented in the text or supplementary material.

## Supplementary Materials

The following supporting information can be downloaded at: https://www.mdpi.com/article/10.3390/diagnostics13030377/s1, S1: records after duplicates removed.
